# Safety and disease response to MEDI-551, an anti-CD19 antibody, in chronic lymphocytic leukemia patients previously treated with rituximab

**DOI:** 10.1186/2051-1426-1-S1-P43

**Published:** 2013-11-07

**Authors:** Andres Forero, Mehdi Hamadani, Thomas Kipps, Michelle Fanale, Antonio Cuneo, Jaime Perez de Oteyza, Douglas Gladstone, Marc Andre, Naresh Bellam, Trishna Goswami, Ramy Ibrahim, Amy Schneider, Meina Liang, Steven Eck, Nairouz Elgeioushi, Ronald Herbst, Bruce D Cheson

**Affiliations:** 1 University of Alabama at Birmingham, Birmingham, AL, USA; 2 West Virginia University, Morgantown, WV, USA; 3 Moores UCSD Cancer Center, San Diego, CA, USA; 4 University of Texas, Houston, TX, USA; 5 Azienda Ospedaliera Universitaria Arcispedale Sant'Anna, Ferrara, Italy; 6 Centro Integral Oncologico Clara Campal, Madrid, Spain; 7 Johns Hopkins University, Baltimore, MD, USA; 8 CHU Mont-Godinne, Yvoir, Belgium; 9 MedImmune, Gaithersburg, MD, USA; 10 MedImmune, Gaithersburg, CA, USA; 11 Georgetown University Hospital, Washington DC, MD, USA

## Background

The expression of CD19 on chronic lymphocytic leukemia (CLL) cells offers a novel therapy for relapsed CLL patients (pts) previously treated with rituximab. MEDI-551 is an affinity-optimized anti-CD19 Ab with enhanced Ab-dependent cellular cytotoxicity (ADCC) effector function.

## Methods

Response and toxicity of single-agent MEDI-551 in multiply relapsed CLL pts with prior rituximab therapy was assessed in a phase 1/2 (ph 1/2), open-label, dose-escalation and expansion study. Combination therapy was assessed in an ongoing phase 2 (ph 2) study comparing MEDI-551 or rituximab+bendamustine in relapsed/refractory CLL pts. For the ph 1/2 study, B cell depletion was assessed with flow cytometry and BAFF biomarker analysis; response was assessed using the 2008 IWG criteria.

## Results

In the ph 1/2 study, 26 CLL pts received ≥1 dose of MEDI-551. In the ph 2 study, 44 pts received study drug as of 20Mar2013. Loss of CD19 detection due to depletion and/or occupancy with MEDI-551 was rapid and apparent after cycle 1. B cell depletion occurred 1 day after dose 1 and was associated with increased serum BAFF concentrations. In the ph 1/2 study, of 21 MEDI-551-treated CLL pts evaluable for response, 5 achieved partial remission and 13 had stable disease. Commonly reported adverse events (AEs) in MEDI-551 pts were infusion-related reactions (IRRs; 62%), nausea (23%), pyrexia (23%), and neutropenia (23%) in the 26 ph 1/2 pts; in the 29 ph 2 pts, they were nausea (62%), IRRs (31%), pyrexia (28%), chills (28%), and fatigue (28%). 11 pts had ≥ grade 3 AEs in the ph 1/2 study and 16 in the ph 2. Common treatment-related AEs: IRRs (58%) and nausea (12%) in the ph 1/2; nausea (52%), IRRs (28%), chills (24%), and fatigue (24%) in the ph 2. Three treatment-unrelated AEs of general health deterioration (ph 1/2), subarachnoid hemorrhage (ph 1/2), and sepsis (ph 2), resulted in death.

## Conclusions

MEDI-551 as a single agent demonstrated B-cell depletion, increased serum BAFF levels, clinical activity, and an acceptable risk-benefit profile in relapsed/refractory CLL pts. Preliminary results of the ongoing ph 2 study of MEDI-551+bendamustine demonstrated an acceptable safety profile.

